# Obesity stigma as a globalizing health challenge

**DOI:** 10.1186/s12992-018-0337-x

**Published:** 2018-02-13

**Authors:** Alexandra Brewis, Cindi SturtzSreetharan, Amber Wutich

**Affiliations:** School of Human Evolution and Social Change, Arizona State University, 900 S. Cady Mall, Tempe, AZ 85287-2402 USA

**Keywords:** Stigma, Weight, Obesity, Overweight, Global health, Globalization, Public health interventions

## Abstract

**Background:**

Based on studies conducted in the global north, it is well documented that those who feel stigmatized by overweight/obesity can suffer extreme emotional distress, be subject to (often legal and socially-acceptable) discrimination, and adjust diet and exercise behaviors. These lead to significant negative health impacts, including depression and further weight gain. To date, weight-related stigma has been conceptualized as a problem particular to the highest income, industrialized, historically thin-valorizing societies like the US, Australasia, and Western Europe.

**Main body:**

There is limited but highly suggestive evidence that obesity stigma is an emergent phenomenon that affects populations across the global south. Emergent evidence includes: implicit and explicit measures showing very high levels of weight stigma in middle and low-income countries, complex ethnographic evidence of widespread anti-fat beliefs even where fat-positivity endures, the globalization of new forms of “fat talk,” and evidence of the emotional and material damage of weight-related rejection or mistreatment even where severe undernutrition is still a major challenge.

**Conclusion:**

Recognizing weight stigma as a global health problem has significant implications for how public health conceives and implements appropriate responses to the growing “obesity epidemic” in middle and lower income settings.

## Background

Weight-related stigma is well established as a pervasive feature of societies in the global north, such as North America, Australasia, and Western Europe. Embedded in cultural valorization of personal effort and individual responsibility, fat bodies symbolize negative traits like laziness and lack of self-control [1]. In such anti-fat contexts, people living with obesity report high attendant levels of systematic discrimination. This manifests directly as teasing, bullying, or being socially ignored or discounted. Indirectly, weight discrimination manifests as reduced education, career, and economic opportunities and success across the lifespan [2].

Based on studies published to date, weight stigma clearly matters for health [3]. For one, it changes health-relevant behaviors for the worse. For example, feelings of weight stigma trigger disordered eating and exercise avoidance -- even when people are desperately trying to lose weight. Feelings of being judged as “fat” are highly emotionally distressing in themselves, and accordingly weight-related stigma predicts higher risk of depression, anxiety, and suicidality. The stress effects of the stigma also likely places people at greater risk of inflammation and chronic disease, as does living with other forms of overt discrimination.

Weight stigma embedded in medical and public health practice can also contribute to the health damage. Medical professionals consistently display high levels of anti-obesity bias, assume obesity suggests patient non-compliance, and admit they would prefer to avoid dealing with obese patients at all. Accordingly, larger patients receive lower quality care, and expecting or fearing negative judgments and mistreatment in clinical settings discourages larger patients from seeking further care.

Public health messages around obesity may also act to reinforce the cultural preoccupations of obesity as individual responsibility and the perpetuate weight stigma. Usually the shaming and blaming is inadvertent, but implied. For example, the Strong4Life campaign in the US emphasized that “fat prevention begins at home and the buffet line,” and in Australia “child obesity is child abuse” was deployed as a campaign message. Yet, some campaigns have been built explicitly on the idea that disproven idea that stigma is a tool that might help motivate weight loss. One proposed strategy in the UK, drawing from anti-smoking successes, was to place pictures of sick, obese bodies onto junk food to scare people away from buying and eating it.

From a public health perspective, also concerning is the growing evidence that all this pervasive weight stigma in itself promotes weight gain, especially for those who are already clinically-defined as obese. There are several different theorized pathways linking weight stigma to population level weight gain, including its influence on the proposed mediators of stress as physiologically damaging, discrimination as a barrier to accessing health care and health-relevant resources, and feeling of judgments leading to weight-positive behavior change. There is growing suggestion it may in fact act as significant, if basically unrecognized, driver of population level weight gain and the obesity epidemic itself.

Accordingly, there is a nascent but energetic push to raise awareness that public health messages around obesity prevention can inadvertently promote stigma and thus ultimately undermine anti-obesity efforts and inadvertently create other related health disparities. This includes recommendations to create anti-obesity campaign messages that do not aggravate weight stigma, and to address weight stigma reduction as part of anti-obesity efforts in the US, the UK, and several other advanced industrialized nations with high rates of obesity. Other anti-stigma efforts promoted by academics, medical experts, and activists include pushing back against negative stereotyping in mainstream media and efforts to include obesity as a class covered by anti-discrimination legislation.

In terms of weight stigma and global health, conventional wisdom suggests that weight stigma is essentially a “first world problem.” Anthropological cases are often cited as demonstrating that many or most human groups view large and fat bodies in positive terms, such as symbolic of goodness, beauty, care, and effort [1]. This conclusion is based in decades of solid, high quality ethnographic enquiry. For example, an extended ethnography of Jamaican views of bodies, conducted in the 1980s, plump bodies are described as sexy, healthy, happy, and loved. Thinness, by contrast, is associated with meanness and social isolation. Another detailed study of a semi-nomadic Azawagh Arabs in Niger at around the same time describes the great lengths women – and their families – go to in order to develop the soft, rounded and very fat bodies that are seen as most godly, loved, and marriageable.

As yet, there has been almost no consideration of links between weight stigma and anti-obesity efforts in the global south. But many countries are right now moving forward with public health campaigns to address obesity, and the early signs are that messages appear to similarly link to notions of blame or shame as they have in many prior campaigns designed for and implemented in the global north. For example, in 2015 anti-obesity legislation in Puerto Rico proposed large fines that punished parents if their children did not lose weight.

As governments in the global south roll out large-scale and costly anti-obesity efforts, it is thus timely and important to ask two basic questions from an evidence-based position: *Is there any evidence of weight-related stigma in middle and lower income countries? And, if so, does experiencing it similarly damage the wellbeing of individuals?* Herein we draw together recent and disparate anthropological, linguistic, and other evidence to show there is growing reason to suspect that anti-fat ideas, and their negative health consequences, may be a rapidly emerging across the global south – including in cultural settings where fat-positivity was previously the norm.

If weight stigma is growing in the global south, recognition of this as a *global* health challenge is crucial. This is because obesity itself is now a truly global phenomenon: there are currently numerically more obese people in India and China than anywhere else, and many of the “fattest” nations by percentage are in the Middle East, South Pacific, or Africa. If weight stigma is evident and impacts people emotionally in such places, it suggests a much-needed research agenda on the implications of globalizing obesity stigma for obesity prevention and treatment in middle and lower income countries. And, it has significant potential practical implications for how global public health should understand and respond to obesity, including identifying vulnerabilities, formulating policy, intervention design, and the need to recognize and track weight-related stigma with a goal of making global anti-obesity efforts more effective.

## Ethnographic evidence of rising obesity stigma

Based on very recent ethnographic studies, increasingly anthropologists are reporting powerful new anti-fat social norms, not evident in the same locales even 5–10 years ago [4]. For example, ethnographic research in Fiji conducted in the 1980s–1990s described the high value placed on the well cared for, large body by the community; by the mid 2010s large bodies are increasingly viewed as uncontrolled and undesirable. In the early 2000s, ethnographic research identified that Belizean women understood large curvy bodies as especially attractive. But participant observation in the same locales in 2015 suggests a rapid, recent switch to anti-fat sentiments whereby the large body was seen as a social and economic liability. For example, publically-veiled young women in the United Arab Emirates now suggest fatness is abhorrent because it blocks upward mobility. In a word-attribution study in a single food-insecure community in Guatemala, school children showed aversion to overweight (and also very thin) bodies, labeling with adjectives such as lazy and ugly [5]. In all these ethnographic cases, anthropologists with long-term local familiarity are concluding that people are expressing new, real cultural concerns around fat bodies, and these are creating feelings of being judged, rejected, and ashamed.

Similar findings emerged in a cultural survey conducted across diverse country sites in 2010, using the comparative approach offered by cultural consensus analysis testing for normative statements about bodies, such as *people are fat because they are lazy.* Samples from sites in American Samoa, Mexico, and Paraguay presented higher prevalence of such anti-fat norms than those collected in the US, UK, and New Zealand [6].

The ethnographic cases also indicate that adoption of new anti-fat norms need not eradicate pro-fat ones. People can be pro-fat and anti-fat, with the switch depending on immediate social context. For example, recent research in Western Polynesia suggests that even while people have anxieties around large bodies, in some specific contexts of daily lives like church or during feasts that large bodies remain valued and expected ( [7]). That is, people can hold dual models of fat – both pro- and anti-fat norms – simultaneously.

## Linguistic evidence of fat talk

A very small set of available studies focused on spoken and written language evidence also suggests that anti-fat ideas are globalizing. Particularly, one recent of global Yahoo Answers searches of “Am I fat?” showed that the same concern is being regularly typed into search engines in many countries [8]. Linking these “Am I fat?” queries to same-user online searches around “bullying,” the study concluded these searches commonly co-occurred across all of those reporting regardless of country (although noting the actual countries are not specified in the study report).

“Fat talk” is a particular discursive interaction, most studied to date in the US. Fundamentally fat talk is best seen as an interactional achievement among solidary speakers. On the one hand, it is a language-based cue of anxieties about the self in relation to concerns of excess body weight and size; on the other hand, it demonstrates the potential for reaffirmation of social affiliation in relation to peers. An example of this discourse is the “I’m so fat”: “No, you aren’t” interaction reported between young women in American schools and universities.

Our own most recent unpublished field data collection in 10 countries suggests that some form of this fat talk is evident in multiple international sites. For example: in Japan, when prompted with an image of a woman (a 5 on the ‘Stunkard’ pictorial body image figure scale [9]) trying on a one-piece bathing suit and asking “Does this make me look fat?” the overwhelming majority of female respondents replied “No, not at all!”; likewise, in Paraguay when provided with the same prompt, women replied with “No, you look good.” Preliminary analysis reveals that in all 10 sites, at least in urban contexts, both sides of fat talk discursive interaction are easily produced without hesitation.

## Evidence of psychometric assessment of anti-fat bias

Much of the evidence of explicit weight sigma (in psychological terms, capturing conscious bias) in the global north has been collected using standardized, validated self-report measures, such as the Attitudes to Obese People (ATOP) scale. Very few of these have been applied in field studies in the global south. Small studies in rural Dominica, Bolivia, and Paraguay using the ATOP have reported extremely high levels of anti-fat attitudes. Data collected from 338,121 self-selected internet users from 71 nations that completed at Project Implicit’s website showed considerable variability across nations in weight-stigma based on people’s self-reports on the ATOP scale. The highest average levels were in responses from website visitors from countries from across both the global north and global south: South Korea, France, Russia, Bolivia, and Romania [10].

**Fig. 1:**
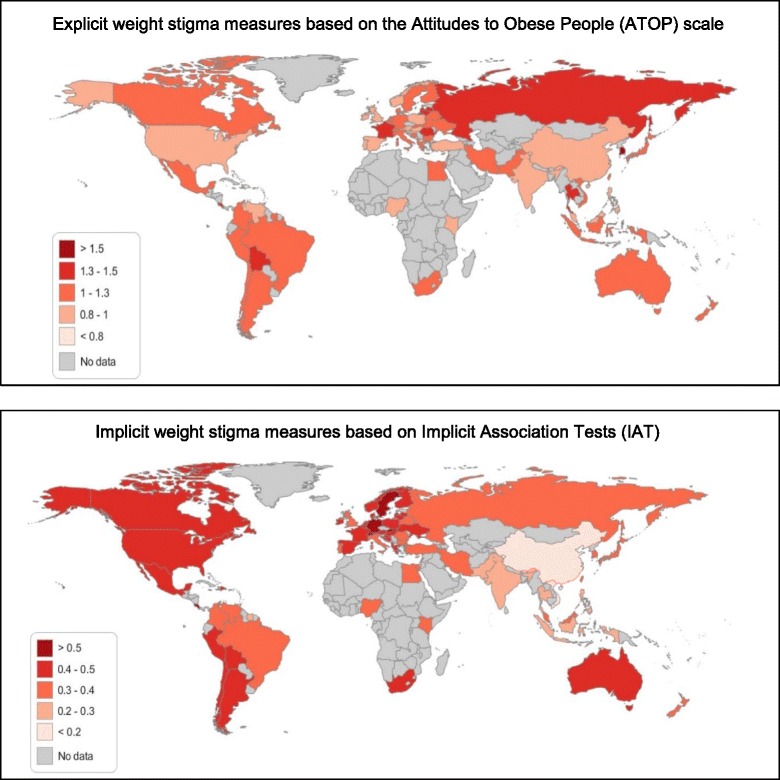
Chloropleths mapping average explicit and implicit weight-bias by country from responses provided by self-selected visitors to the Project Implicit website. Higher scores suggest greater weight stigma. Figures adapted by the authors from tabular data provided in Marini et al. (2013) online supplementary appendix [10]

Implicit stigma (in psychological term, unrecognized biases), can be captured in implicit association tests (IATs). The same Project Implicit dataset suggested that countries with higher levels of obesity and higher Gross Domestic Product (GDP) were presented with higher average levels of implicit stigma (also see Fig. 1). By comparison these factors did not appear to influence country-level measures of *explicit* stigma, as measured on the standard ATOP scale.

Relatedly, data from sites in Bolivia and Paraguay have shown implicit bias measures on IATs can be low, importantly, even as explicit measures on the ATOP are high [11]. The same study showed that, by comparison, US students were on average higher on implicit and lower on explicit measures of weight stigma. The authors suggest concerns for political correctness might help account for this difference, whereby young highly educated people in US were accustomed to monitoring and managing how they responded on the interview scales to meet competing norms for avoiding openly discriminating statements, but not on IATs (which are harder to modulate in this way). This suggested that overt discrimination around weight would be more common in the Global South sites, because there was less of a social taboo around expressing high levels of anti-fat comments or teasing.

## Evidence that growing obesity stigma damages health

A crucial concern for global heath is whether these emergent indications of weight stigma in the global south have negative health effects as has been documented in the US-based literature in particular. The best current evidence is analysis of a large, population-representative survey with reproductive age women in Guatemala. It found that weight-based teasing is commonly reported, with a similar effect on depressive levels as living with domestic violence or food insecurity or through the civil war [12]. Importantly for a country where malnutrition and attendant stunting remains common, the depressive effects were seen in women who reported being teased for being underweight just as much as overweight. Recent ethnographic studies from the United Arab Emirates, Belize, and Fiji also suggest that feelings of weight stigma contribute to potentially damaging patterns of disordered eating [4].

## Globalizing obesity stigma: Implications, challenges, and opportunities

The (limited) research to date on weight stigma in middle and lower income countries suggests: (a) anti-fat norms are now globally spread, (b) this shifted happened very recently, (c) in some sites in the global south that overt fat-shaming may be more socially acceptable (normative) than in the global north, (d) that this shaming can be highly distressing to people, and thus (e) potentially health-damaging.

Based on this set of suggestions, in this final section we outline key points for an agenda that would move our currently very limited knowledge about weight stigma’s implications for global health forward.

### Apply tools to identify and track weight-related stigma across culturally diverse settings and through time

We need a wider evidence base to track if and how weight-related stigma is manifesting across diverse locations, including how anti-obesity efforts impact it. Currently, to our knowledge, there have been have no large-scale studies based on data collected in situ using culturally-adapted versions of standardized, validated tools like the ATOP and IAT. For the localized studies that have been done using such tools, the range of countries represented and sample sizes are both small.

### Establish the negative effects of anti-fat norms on mental and physical health

There is very little direct study to date on the point of whether these new anti-fat norms are emotionally damaging outside of the global, industrialized north. The depth and power of these norms in places like the US and UK is deeply embedded in powerful cultural ideals around work, productivity, and responsibility. Ethnographic studies are needed to clarify the conditions under which new globalizing body norms manifest in feeling of being judged, rejected, or otherwise stigmatized or not. For example, if the suggestion that dual body models create enough ambivalence to be emotionally or health-protective, this suggests that adding or supporting alternative body models/norms rather than trying to eliminate stigmatizing ones could be easier and effective as an anti-stigma strategy.

Consider the case of fat talk. Linguistic analyses can identify if the prominent rituals of fat talk seen in the West are as evident and meaningful in other parts of the word. Interview and focus group studies can identify if public health messaging is interpreted similarly across sites. Experimental studies (or using existing interventions as natural experiments) could identify how norms might change health-seeking or other health relevant behaviors, such as desire to exercise. This may help suggest where anti-stigma efforts should be focused, and provide insights as to how anti-stigma efforts in general can be designed to have greater impact.

### Clarify the possible role of public health messaging and obesity campaigns in advancing and reinforcing anti-fat norms and weight stigma

A major legitimate concern needing direct investigation is whether anti-obesity policies and practices being conducted in the service of public health might in themselves be contributing weight-related stigma in middle and lower income countries. That is, a greater public expression of concern about weight (e.g., policy debate about obesity, soda taxes, public health efforts, advertisements for diet food) might be part of the reason norms are changing, permitting or even generating new overt, damaging forms of shaming and discrimination related to being obese. We suspect that, placed in a cross-cultural lens, the relationship between public health weight loss messaging and weight stigma is likely to be increasingly complicated. Consider preliminary research with Korean women that indicates that judgmental weight loss messaging is associated with *less* body dissatisfaction [13].

### Widen the construct of weight stigma to accommodate discrimination and exclusions based on under-nutrition and thin-aversion, and the complexities of co-occurrences of weight positive and weight negative social norms

Currently, weight-related stigma research conducted in North America, Western Europe, and Australasia focuses almost entirely on large, fat bodies as the form that socially problematic. Research and interventions related to weight stigma in the global south may benefit from re-conceptualizing weight stigma to include underweight as much as overweight bodies -- that is, to encompass all bodies defined as ‘not normal’. Moreover, it seems the relations between weight stigma and ill-health may be particularly complicated in places with a long history of fat-positivity. We need to begin to consider what it means for stigma when fat-negative and fat-positive ideas exist in dual models of healthy bodies. This may be especially important to untangling the possible consequences of weight stigma in the growing number of countries (such as Mexico, China, or India) where anti-obesity efforts are being rolled out at the same time that undernutrition programs still remain a priority.

## Conclusion

In the US and other parts of the global north, the ‘epidemic’ of weight stigma grew unrecognized for decades, with apparently complicity in medical and public health norms and practices that reflected the cultural view of obesity as a moral failing. This has had profound negative emotional and health effects on millions. As governments in the global south increasingly roll out large-scale and costly anti-obesity efforts, it is timely and important to ask two basic questions from an evidence-based position: Is weight-related stigma evident in middle and lower income countries? And, if so, is it similarly damaging to the wellbeing of individuals? On the basis of early and mostly preliminary studies, we propose there is reasonable evidence that the answers to both questions may be ‘yes.’ Given what has been learned from prior mistakes that have slowed or scuttled anti-obesity efforts to date, we have the opportunity to ensure that, as anti-obesity efforts globalize, such campaigns do not promulgate further new overt, damaging forms of shaming and discrimination related to being obese—in exactly the forms that ultimately can undermine public health efforts.
